# Effect of intraoperative neuromuscular blockade on postoperative sore throat and hoarseness in patients undergoing spinal surgery: a prospective observational study

**DOI:** 10.1038/s41598-020-71897-9

**Published:** 2020-09-09

**Authors:** Dongwook Won, Jee-Eun Chang, Hyerim Kim, Jung-Man Lee, Yoomin Oh, Jin-Young Hwang

**Affiliations:** 1 Department of Anesthesiology and Pain Medicine, SMG-SNU Boramae Medical Center, Boramae-ro 5, Dongjak-gu, Seoul, 07061 Republic of Korea; 2 Department of Anesthesiology and Pain Medicine, Seoul National University Hospital, Seoul, Republic of Korea; 3 College of Medicine, Seoul National University, Seoul, Republic of Korea

**Keywords:** Medical research, Signs and symptoms

## Abstract

Intraoperative neuromuscular blockade affects the resting tension between the vocal cords. We assessed the effect of neuromuscular blockade on postoperative sore throat and hoarseness following tracheal intubation in patients undergoing lumbar spinal surgery in the prone position. Altogether, 99 patients were included; 50 patients did not receive neuromuscular blockade, and 49 patients received moderate neuromuscular blockade during the maintenance of anesthesia. Neuromuscular blockade was performed depending on the use of intraoperative neurophysiological monitoring. The number of intubation attempts, time to achieve tracheal intubation, and duration of intubation were recorded accordingly. The incidence and severity of postoperative sore throat and hoarseness was assessed at 1, 6, and 24 h after surgery. The overall cumulative incidence of postoperative sore throat (60% vs. 59%, respectively; *P* = 1.000) and postoperative hoarseness (68% vs. 61%, respectively; *P* = 0.532) did not differ between the no neuromuscular blockade and moderate neuromuscular blockade. The incidence and severity of postoperative sore throat and hoarseness was also not different between the moderate and no neuromuscular blockade at each time point after surgery. Nevertheless, the incidences of postoperative sore throat and hoarseness were quite high. Further studies investigating strategies to alleviate them are warranted accordingly.

## Introduction

Neuromuscular blocking agents are routinely used to facilitate tracheal intubation during induction of anesthesia and are commonly used to improve surgical conditions during the maintenance of general anesthesia. However, there have also been several concerns about their effectiveness in improving surgical conditions and side effects, including delayed recovery, residual paralysis, and hypersensitivity reactions^1–4^.

Neuromuscular blocking agents act on skeletal muscles, including the laryngeal adductor muscles that close the glottis, and decrease the resting tension between the vocal cords^5^. Postoperative sore throat and hoarseness is an undesirable complication following tracheal intubation, which is attributed to irritation and inflammation related to the presence of a tracheal tube^6^. Under neuromuscular blockade (NMB), the vocal folds are abducted; thus, mechanical stimulus and contact pressure between the tracheal tube and vocal folds may be alleviated, that could affect postoperative sore throat and hoarseness. However, it is unclear whether intraoperative NMB affects postoperative sore throat and hoarseness following tracheal intubation.

Since, NMB is generally required for the maintenance of general anesthesia, we considered the randomized use of NMB for research purposes as inappropriate. The present study was hence, based on patients undergoing lumbar spinal surgery with or without intraoperative neurophysiological monitoring because NMB is performed or avoided in these patients, depending on the requirement for intraoperative neurophysiological monitoring. We observed the effect of intraoperative NMB on postoperative sore throat and hoarseness in patients undergoing lumbar spinal surgery with or without intraoperative neurophysiological monitoring.

## Methods

### Patients

After obtaining written informed consent, adult patients who were scheduled to undergo elective lumbar spinal surgery were recruited. Patients with a predicted or known difficult airway, neuromuscular diseases, hepatic or renal dysfunction, or anatomical abnormalities or diseases in the larynx, pharynx, or neck and those requiring postoperative mechanical ventilation were excluded. Patients with Cormack and Lehane grade 3 or 4 during direct laryngoscopy were also excluded from the study.

### Anesthesia and study protocol

No premedication was administered to the patients. Standard intraoperative monitoring included electrocardiography, non-invasive and invasive arterial pressure, pulse oximetry, and gas analyses. Anesthetic depth was monitored using the bispectral index (BIS SENSOR; Covidien, Boulder, CO, USA). Anesthesia was induced by continuous infusion of propofol and remifentanil, and rocuronium 0.6 mg/kg was administered to establish NMB. After confirming the loss of consciousness, neuromuscular function was monitored by train-of-four (TOF) stimulation using an acceleromyograph (TOF-WATCH SX, Organon Ireland Ltd., Dublin, Ireland) at the level of the ulnar nerve. At a TOF count of zero, tracheal intubation was performed under direct laryngoscopy using wire-reinforced tracheal tubes (internal diameter of 7.5 mm for men and 7.0 mm for women)^7^ by an experienced board-certified anesthesiologist. A rigid stylet was used inside the tracheal tube to make a curvature. Tracheal intubation was confirmed by square waves on capnography. The intracuff pressure was monitored every 30 min using a handheld aneroid manometer (VBM MEDIZINTECHNIK GMBH, Sulz am Neckar, Germany) and adjusted to 25 cmH_2_O^8^. Body temperature was monitored using an esophageal temperature probe and maintained at approximately 36 °C using a forced-air warmer. After induction of anesthesia, the patient’s position was changed from supine to prone. Once neurophysiological monitoring was planned, the needle electrodes were placed in the patients accordingly. Anesthesia was maintained using continuous infusion of propofol and remifentanil to conserve the bispectral index between 40 and 60. Mechanical ventilation was performed using a volume-controlled mode with a tidal volume of 8 mL/kg, and the adjusted respiratory rate was applied to maintain normocapnia^7^. During the maintenance of anesthesia, the use of NMB was determined by whether or not neurophysiological monitoring was performed on the patient. The use of intraoperative neurophysiological monitoring was determined at the surgery planning stage. In clinical practice, NMB is avoided when intraoperative neurophysiological monitoring is performed. Therefore, no additional rocuronium was administered after the induction of anesthesia in patients in whom intraoperative neurophysiological monitoring was performed. If intraoperative neurophysiological monitoring was not scheduled, moderate NMB was achieved as the usual clinical practice. The degree of NMB was monitored every 15 min, and additional rocuronium 0.15 mg/kg was administered, if required, to maintain the TOF count at 1 or 2. The infusion of propofol and remifentanil was stopped at the time of skin suture. The patient’s position was changed from prone to supine after applying a surgical dressing.

Residual NMB was reversed using pyridostigmine and glycopyrrolate in the moderate NMB group. Extubation was performed when the patients fully recovered. An intravenous patient-controlled analgesia (PCA) device was applied at the end of the surgery if requested^7^. This study was conducted in accordance with relevant guidelines and regulations.

### Outcomes

During tracheal intubation, the Cormack and Lehane grade, number of attempts for tracheal intubation, and time taken for tracheal intubation were recorded. The duration of tracheal intubation and total propofol and remifentanil administration were also recorded.

Sore throat and hoarseness was assessed at 1, 6, and 24 h after surgery by investigators unaware of the group allocation^8^. The severity of sore throat was evaluated using a 100-point numerical rating scale scored from 0 (no pain) to 100 (worst pain imaginable). The results were classified as follows: 0 (none); 10 − 30 (mild); 40 − 60 (moderate); and 70 − 100 (severe)^9^. Hoarseness was defined as a voice quality change different from the preoperative voice and graded as follows: none (no hoarseness), mild (recognized by the patient), moderate (obvious to the investigator), and severe (aphonia)^10^. The overall postoperative sore throat or hoarseness cases included patients who complained of sore throat or hoarseness over the 24-h evaluation period^8^. PCA fentanyl consumption and analgesic medications administered within 24 h after surgery were recorded accordingly.

### Statistical analysis

Based on a reported incidence of postoperative sore throat of 40%^11^, a sample size calculation was performed assuming a clinically significant difference in the postoperative sore throat of 30% depending on NMB, and 48 patients per group were required at a significance level of 95% and a power of 80%. Considering the possible dropouts, 53 patients per group were finally enrolled.

SPSS version 20 (IBM Corporation, Armonk, NY, USA) was used to perform the statistical analyses. Data normality was tested using the Shapiro–Wilk test. Data are expressed as patient numbers (%), or mean ± SD. Differences in continuous variables between the two groups were compared using Student’s t-test or Mann–Whitney U-test. Differences in categorical variables between the two groups were tested using the chi-squared test or Fisher’s exact test. A *P* − value < 0.05 was considered statistically significant.

### Ethical approval

This study was approved by the Institutional Review Board of SMG-SNU Boramae Medical Center (20170605/10-2017-6/063), and was registered at ClinicalTrials.gov (NCT03397797, date of registration: 12th January 2018).

## Results

**Figure 1:**
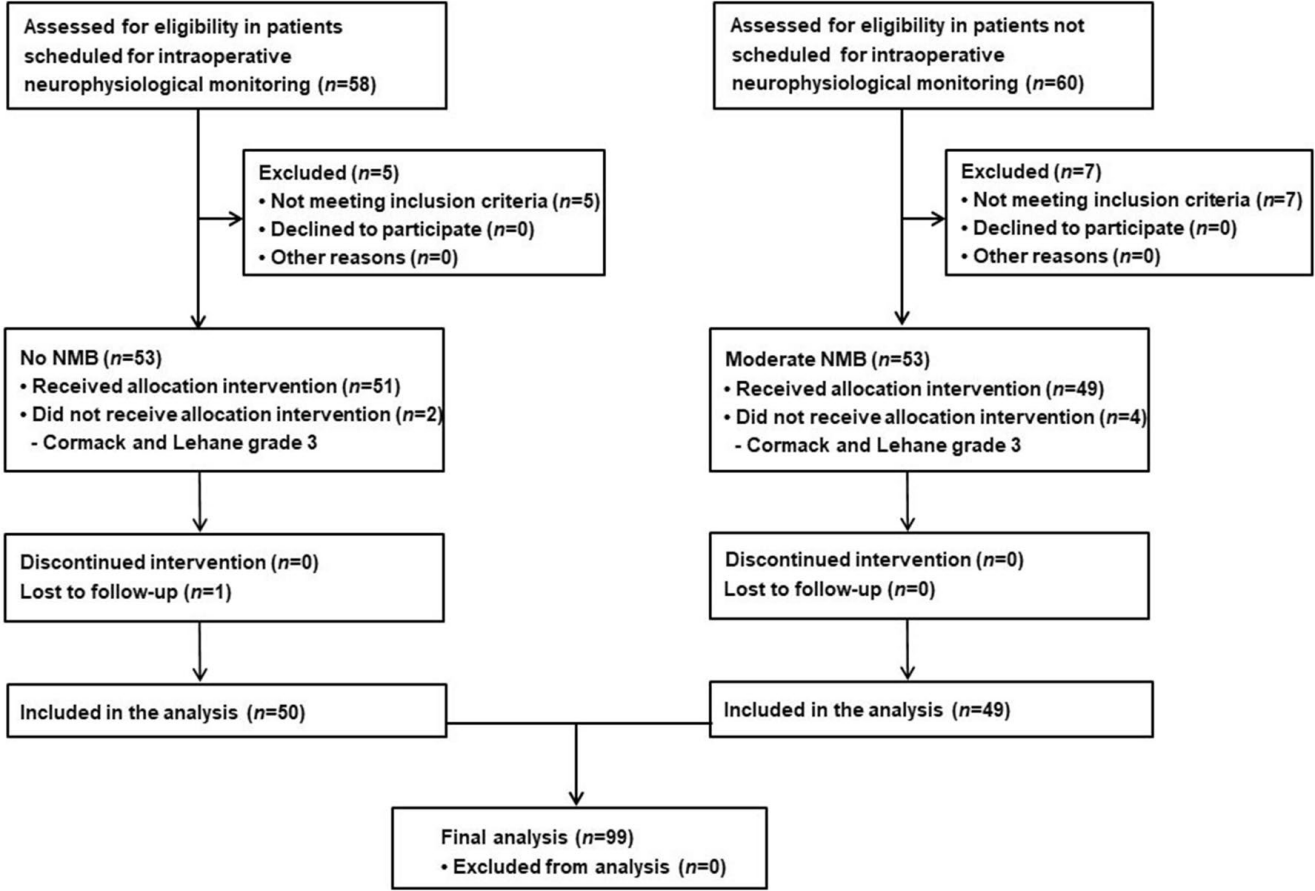
Study flowchart. *NMB* neuromuscular blockade.

Among patients scheduled for intraoperative neurophysiological monitoring, 58 were assessed for eligibility, and five were excluded because they did not meet the inclusion criteria. From these 53 patients, two were excluded due to Cormack and Lehane grade 3 and one patient was excluded because some of their data were missing. Among patients not scheduled for intraoperative neurophysiological monitoring, 60 were assessed for eligibility, and seven were excluded because they did not meet the inclusion criteria. From these 53 patients, four were excluded due to Cormack and Lehane grade 3 on direct laryngoscopy. Ultimately, 99 patients (50 with no NMB and 49 with moderate NMB) were included in the final analysis (Fig. 1).

Patient and surgery-related data are summarized in Table 1. Tracheal intubation attempts, time taken to achieve tracheal intubation, and duration of tracheal intubation were not different between the two groups (Table 2).

**Table 1:** Patient characteristics and surgery-related data.

Factors related to patient and surgery	No NMB (n = 50)	Moderate NMB (n = 49)
Age (years)	66 ± 11	68 ± 8
Sex (M/F)	21/29	20/29
Weight (kg)	63 ± 13	64 ± 9
Height (cm)	160 ± 8	160 ± 9
Total propofol administration (mg)	1778 ± 545	1781 ± 552
Total remifentanil administration (μg)	1507 ± 601	1,467 ± 557
**Postoperative analgesia**		
PCA with fentanyl	49	49
Morphine	2	9
Tramadol	18	23
Acetaminophen	9	5
Ketorolac	11	8
Demerol	5	1
Fentanyl during 24 h after surgery (μg)	404 ± 209	422 ± 198
Duration of surgery (min)	217 ± 96	213 ± 74
Duration of anesthesia (min)	284 ± 91	280 ± 75

Values are means ± SD or number of patients.

*NMB* neuromuscular blockade, *PCA* patient-controlled analgesia.

**Table 2:** Factors related to tracheal intubation.

Factors related to tracheal intubation	No NMB (n = 50)	Moderate NMB (n = 49)	*P*-value
Cormack and Lehane grade (1/2)	27/23	27/22	1.000
Number of attempts (1/2)	50/0	48/1	0.495
Time for tracheal intubation (sec)	28 ± 7	27 ± 6	0.936
Duration of tracheal intubation (min)	277 ± 91	274 ± 75	0.869

Values are means ± SD or number of patients (%).

*NMB* neuromuscular blockade.

**Figure 2:**
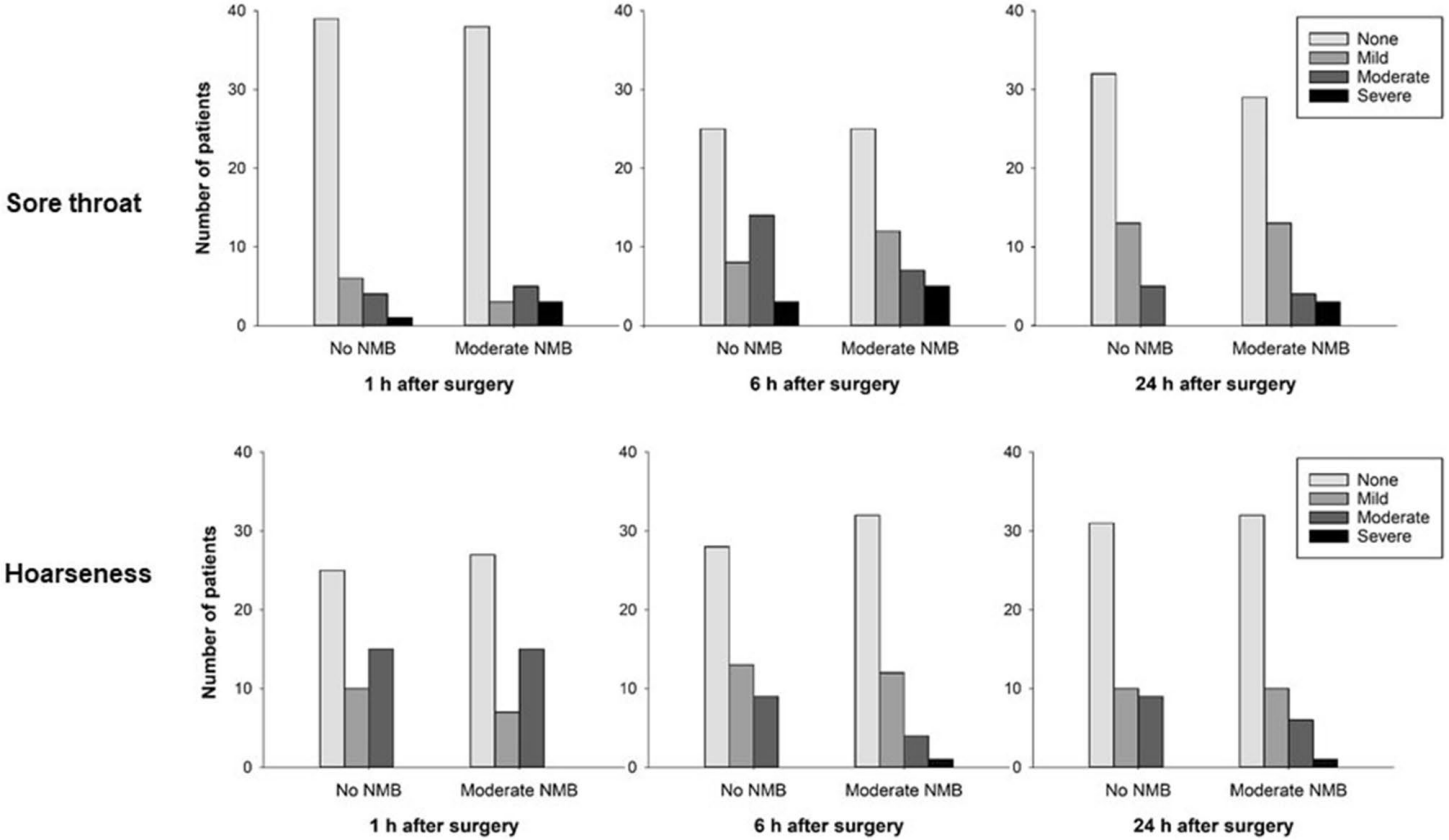
Incidence and severity of postoperative sore throat and hoarseness. *NMB* neuromuscular blockade.

**Figure 3:**
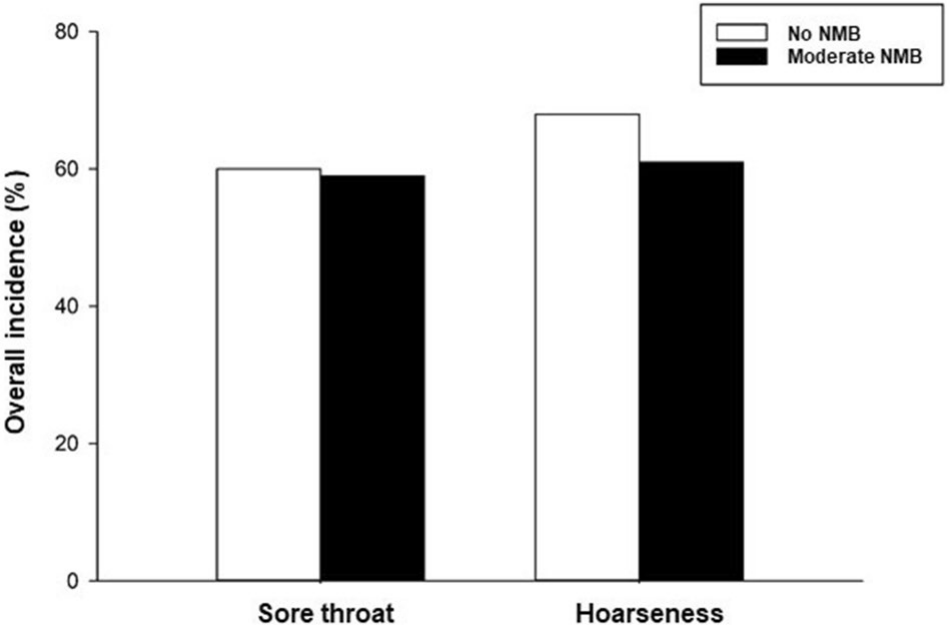
Overall incidence of postoperative hoarseness sore throat and hoarseness. *NMB* neuromuscular blockade.

The incidence and severity of postoperative sore throat and hoarseness are shown in Figs. 2 and 3. The overall incidence of postoperative sore throat did not differ between the no NMB and moderate NMB groups (60% vs. 59%, respectively; *P* = 1.000). Eleven patients in the no NMB group presented sore throat at 1 h, and three of these 11 patients did not experience it at 6 h. In this group, seventeen patients complained of a new sore throat at 6 h, and one patient complained of sore throat at 24 h. In the moderate NMB group, eleven patients experienced sore throat at 1 h, and three of these 11 did not have sore throat at 6 h. Then, sixteen patients had a new sore throat at 6 h after surgery, and two patients reported a new sore throat at 24 h. The incidence and severity of postoperative sore throat at each time point were not different between the two groups. The overall incidence of postoperative hoarseness was also not different between the no NMB and moderate NMB groups (68% vs. 61%, respectively; *P* = 0.532). The incidence and severity of postoperative hoarseness was not different at 1, 6, and 24 h after surgery between the two groups. During assessment, 25 patients in the no NMB group and 22 in the moderate NMB group experienced hoarseness at 1 h; however, at 6 h after surgery, 10 in the no NMB group and 11 in the moderate NMB group had no more hoarseness. Seven patients in the no NMB group and six in the moderate NMB group reported a new hoarseness at 6 h. Furthermore, three patients in the no NMB group and two in the moderate NMB group had a new hoarseness at 24 h after surgery.

## Discussion

This study showed that NMB does not affect the incidence and severity of postoperative sore throat and hoarseness in the 24-h evaluation period after lumbar spinal surgery. The overall incidence of postoperative sore throat was 60% and 59% with no NMB and moderate NMB, respectively. Similarly, the overall incidence of hoarseness after surgery was 68% and 61% with no NMB and moderate NMB, respectively.

Postoperative sore throat is a common complication following tracheal intubation, with reported incidences of 40 to 62%^11,12^, and leads to patient discomfort and dissatisfaction during the recovery period. It is affected by several factors including the size of tracheal tubes, intracuff pressure, the use of a rigid stylet, duration of surgery, and movement of tracheal tubes during positional changes^12–14^. Postoperative sore throat and hoarseness following lumbar spinal surgery can be expected to be high due to the routine use of a rigid stylet for tracheal intubation with wire-reinforced tubes and movement of tracheal tubes during positional changes between the supine and prone positions^14,15^. It has been reported that 92% of patients had a displacement of tracheal tubes and 86% of patients had changes in intracuff pressure after the supine-to-prone position change^15^. Moreover, lumbar spinal surgery is performed with the patient in the prone position; as such, upper airway edema can occur due to local compression and venous or lymphatic obstruction from neck rotation or hyperflexion^16^.

A few studies have investigated the effect of NMB on intraoperative and postoperative outcomes during spinal surgery^2,3^. They mainly evaluated surgical conditions and recovery profiles according to the depth of NMB, but did not address postoperative sore throat and hoarseness. To our knowledge, this is the first study to evaluate the effect of NMB on postoperative sore and hoarseness following tracheal intubation in patients undergoing lumbar spinal surgery in the prone position. Our findings—approximately 60% and 59% incidence of postoperative sore throat with no NMB and moderate NMB, respectively,—are consistent with those of previous studies; nevertheless, the incidence of postoperative sore throat was quite high irrespective of NMB in our study. In a previous study^17^, the overall incidence of postoperative sore throat was 62% and the overall incidence of hoarseness was approximately 58% within 48 h after lumbar spinal surgery despite the prophylactic use of dexamethasone, which is known to be effective for reducing postoperative sore throat^18,19^. The incidence of postoperative hoarseness in our study was significantly higher than the previously reported incidence of 28–37%^7,12^. The duration of surgery can affect postoperative sore throat and hoarseness^11^. Multi-level spinal surgery takes a long time when performed on patients in the prone position. Prolonged surgery and large fluid shifts during lumbar spinal surgery in the prone position can cause upper airway edema up to 12 h after extubation^16^. Upper airway edema can cause airway obstruction as well as hoarseness. Therefore, strategies to reduce them should be considered in these patients.

With the use of non-depolarizing neuromuscular blocking agents, the dose required to block the laryngeal adductor muscles is larger than that for the adductor pollicis muscles because the laryngeal muscle is more resistant to neuromuscular blocking agents than other muscles^20^. Intraoperative neuromuscular monitoring is generally performed at the adductor pollicis muscle. In the present study, moderate NMB was performed with neuromuscular monitoring of the adductor pollicis muscle. Therefore, the laryngeal adductor muscles may have partially recovered from NMB during surgery, despite the adductor pollicis muscle exhibiting a TOF count of 1 or 2. According to a previous study investigating the effect of deep NMB on surgical conditions for laryngeal microsurgery^21^, the vocal cords moved less frequently with deep NMB compared to moderate NMB. Therefore, there is a possibility that deep NMB may affect postoperative sore throat and hoarseness following tracheal intubation, although it is not indicated for neurological monitoring during lumbar spinal surgery.

The present study had some limitations. First, this was an observational study and the use of neuromuscular blocking agents was determined depending on whether intraoperative neurological monitoring was performed. Therefore, the investigators were not blinded to the group allocation. However, they adhered to a detailed study protocol. Furthermore, investigators who assessed sore throat and hoarseness after surgery were blinded to the group assignment. Second, patients with a predicted difficult airway were excluded to avoid confounding factors. Therefore, our findings may not be generalizable to them. Third, sore throat and hoarseness were observed only for 24 h after surgery. However, sore throat and hoarseness were alleviated 24 h after surgery. Fourth, we did not directly examine the condition of the vocal cords under no NMB or moderate NMB. Fifth, this study evaluated the effect of NMB on postoperative sore throat and hoarseness in patients undergoing surgery in the prone position. The prone position may cause edema of the pharynx and larynx, thereby affecting the development of sore throat and hoarseness. Therefore, these results may not be applicable to patients undergoing surgery in the supine position.

In summary, this study was performed to evaluate the effect of NMB on postoperative sore throat and hoarseness in patients undergoing lumbar spinal surgery in the prone position, and the incidence and severity of postoperative sore throat and hoarseness were not different between no NMB and moderate NMB. Overall, the incidences of postoperative sore throat and hoarseness were quite high in our study. Hence, further studies investigating strategies to alleviate postoperative sore throat and hoarseness are warranted in these patients.

## Data Availability

The datasets generated and analyzed during the current study are available from the corresponding author upon reasonable request.
